# Current Incisional Negative Pressure Wound Therapy Systems: Technical Characteristics and a Decision Framework for Device Selection

**DOI:** 10.1111/iwj.71074

**Published:** 2026-10-02

**Authors:** Luca Folini, Alfio Luca Costa, Claudio Ligresti, Pasquale Zona, Daniele Brunelli, Vincenzo Vindigni, Franco Bassetto, Carlotta Scarpa

**Affiliations:** ^1^ Department of Neurosciences University Hospital of Padova Padova Italy; ^2^ Department of Plastic and Reconstructive Surgery Maria Pia Hospital Institute Turin Italy

**Keywords:** clinical decision support, closed incision negative pressure therapy, device selection, incisional negative pressure wound therapy, surgical site infection, wound complications

## Abstract

Incisional negative pressure wound therapy has expanded from a niche adjunct to a widely adopted strategy for the prevention and management of wound complications in selected surgical settings. However, available systems differ in pressure profile, fluid handling, dressing interface, and suitability for fragile skin or anatomically challenging incisions, while practical guidance for device selection remains limited. Whether iNPWT should be used selectively is an established question; which system characteristics are relevant once its use has been judged appropriate is not. We conducted a structured narrative review of the literature on incisional negative pressure wound therapy published between January 2013 and September 2026, based on a targeted PubMed/MEDLINE search. Clinical studies addressing primarily closed surgical incisions were reviewed across elective, emergency, and reconstructive settings; conventional negative pressure wound therapy for open wounds was considered only in order to define the boundary between the two indications. Device technical characteristics were extracted separately from manufacturers' instructions for use and clinician guidelines. Evidence was synthesised in relation to clinical indication, incision and patient characteristics, device features, reported outcomes, and treatment‐related adverse events. On this basis, a clinical‐requirement‐first decision framework for bedside device selection was developed, with each component labelled as direct clinical evidence, indirect clinical evidence, documented technical characteristic, or expert clinical consideration. The available evidence supports a selective rather than routine use of incisional negative pressure wound therapy. The greatest clinical value appears in high‐risk incisions and in settings where oedema, superficial fluid accumulation, or limited soft‐tissue protection are likely to contribute to wound morbidity. Reported benefits vary according to procedure type, baseline risk, endpoint definition, and device configuration. Published studies evaluate complete proprietary systems rather than isolated technical variables. The only randomised comparison between two commercial systems identified was small and found no difference between them. Adverse events remain relevant and may limit treatment in fragile skin or when drainage exceeds the fluid‐handling capacity declared for the selected dressing. Incisional negative pressure wound therapy should be considered an adjunct to standard preventive measures, not a universal solution. A structured approach integrating anticipated fluid‐handling requirements, perilesional skin tolerance, seal feasibility over the relevant anatomy, and intended therapy duration may support more rational and transparent device selection once iNPWT has been judged appropriate. Current evidence does not establish a hierarchy among commercially available incisional systems, and the framework should be regarded as structured decision support rather than as a validated algorithm.

## Key Points

- Incisional negative pressure wound therapy (iNPWT) should be used selectively, as an adjunct to standard preventive measures rather than routinely.
- Trials evaluate complete proprietary systems, and current evidence does not establish a hierarchy among commercially available devices.
- Once iNPWT is indicated, device choice can be guided by fluid‐handling need, skin tolerance, seal feasibility and therapy duration; the framework requires prospective validation.

## 1 Introduction

Negative pressure wound therapy applies controlled subatmospheric pressure through a sealed dressing connected to suction. Its proposed effects include oedema reduction, fluid removal, modulation of the wound microenvironment, and support of granulation tissue formation [1, 2, 3]. Wound healing is shaped by systemic and local factors, including metabolic disease, vascular impairment, smoking, and infection, which can delay progression through the normal reparative phases [4, 5]. These biological principles provide the rationale for therapies that modify the local wound environment.

Incisional negative pressure wound therapy extends these principles to primarily closed incisions. The dressing is applied across the incision line and surrounding skin with the aim of stabilising wound edges, shielding the incision from external shear, limiting superficial fluid accumulation, and protecting vulnerable wound margins. This target distinguishes iNPWT from conventional NPWT, which is applied to an open wound bed and depends on a filler interface, cavity management, and active removal of larger fluid volumes. The two applications are not interchangeable, and the present review is confined to primarily closed incisions; open wounds are referred to only where they define the boundary between the two indications. In current practice, iNPWT is used across clean elective procedures, clean contaminated surgery, reconstruction, and trauma. The evidence base is broad but heterogeneous, and reported outcomes vary according to procedure type, baseline risk, device configuration, and endpoint definition [6, 7, 8, 9].

Available studies and consensus documents support a selective rather than routine use of iNPWT, with particular attention to patient risk profile, local incision characteristics, and expected drainage [6, 7, 8, 9, 10]. Which incisions and which patients may benefit is therefore a question that existing indication‐based recommendations already address. A second and largely unanswered question arises at the bedside once iNPWT has been judged appropriate: which technical characteristics of the available systems are relevant to that specific clinical situation. Commercially available systems differ in pressure generation, dressing interface, fluid handling, canister configuration, adhesive technology, portability, and intended wear time, and these differences are not addressed by indication‐based recommendations. The present structured narrative review therefore examines the characteristics of current iNPWT systems, summarises their documented characteristics, and proposes a decision framework that proceeds from the clinical requirements of the incision and the patient to the device characteristics capable of meeting them. The framework is evidence‐informed and is not offered as a validated ranking of commercial systems.

## 2 Materials and Methods

This study was designed as a structured narrative review with development of a decision framework for iNPWT device selection. The narrative approach was chosen to integrate heterogeneous evidence across surgical settings, procedure types, endpoints, and device designs, and to relate technical device characteristics to practical clinical scenarios. The review was not conducted as a systematic review: no protocol was registered, screening was not duplicated by independent reviewers, no study flow diagram was generated, and no formal risk‐of‐bias or certainty‐of‐evidence assessment was performed. The conclusions are limited accordingly and are presented as structured decision support rather than as graded recommendations.

A targeted literature search was performed in PubMed/MEDLINE for articles published from January 2013 to September 2026. Search terms were combined as follows: (‘incisional negative pressure wound therapy’ OR ‘closed incision negative pressure therapy’ OR iNPWT) AND (‘surgical site infection’ OR ‘wound complications’ OR ulcer). Reference lists of the retrieved reviews and consensus documents were screened for additional clinical studies. Clinical studies addressing iNPWT over primarily closed surgical incisions formed the basis of the effectiveness synthesis. Studies of conventional NPWT for open wounds and chronic ulcers retrieved by the same search were retained only to delimit the boundary between the two indications and were not used to support any statement on incisional device selection. Non‐English publications, animal and laboratory studies, editorials, letters, narrative reviews without clinical data, and reports without clear outcome measures related to iNPWT were excluded from the clinical synthesis.

Technical characteristics of the systems discussed were not derived from the clinical literature. They were extracted from the manufacturers' instructions for use, clinician guidelines, and regulatory documentation for each system [11, 12, 13, 14], and are reported as declared specifications. Bench and computational studies were excluded from the clinical synthesis but were used, where explicitly identified as such, to describe technical performance [15, 16]. Declared specifications were not used to infer comparative clinical performance.

From the eligible sources, information was extracted on device characteristics and conditions of use, including pressure settings, dressing configuration, canister configuration, reported surgical or clinical setting, and endpoints such as surgical site infection, wound complications, fluid management, and patient tolerance. For each clinical study, the system used, its nominal pressure and fluid‐management configuration, the comparator, the main endpoint, the principal result, and device‐related adverse events were tabulated (Table 2); fields not stated in the primary publication are recorded as not reported. Findings were synthesised narratively.

The decision framework was constructed to proceed from clinical requirements to device characteristics rather than from clinical scenarios to named systems. Its entry criterion is confirmation that the wound is a primarily closed surgical incision with apposed edges; open wounds, cavities, and lesions requiring a filler interface fall outside the framework and are managed with conventional NPWT. Once iNPWT has been judged appropriate on the basis of patient and procedural risk, the framework identifies four requirements that can be assessed at the bedside and that map onto documented system characteristics: anticipated fluid‐handling requirement, perilesional skin tolerance, seal feasibility over the relevant anatomical contour, and intended therapy duration with the associated mobility requirement. Bleeding risk and other contraindications are treated as gating conditions preceding device selection rather than as selection criteria.

Because no validated cut‐offs exist for these requirements, they were operationalised using observable or functional criteria rather than numerical thresholds. Anticipated fluid‐handling requirement was defined as whether expected drainage is likely to remain within, or to exceed, the fluid‐handling capacity declared for the selected dressing over the intended wear time; where declared, manufacturers express this capacity per unit wound area and per unit time, and it differs between canisterless and canister‐based configurations [11, 12, 13, 14]. Because these data are not provided in directly comparable terms for all systems, this criterion remains qualitative and does not support a quantitative comparison between devices. Perilesional skin tolerance was defined by clinical characteristics associated with susceptibility to medical adhesive‐related skin injury, namely atrophic or oedematous skin, previous irradiation of the operative field, long‐term corticosteroid or immunosuppressive therapy, and any previous adhesive‐related injury or blistering at the same site [28]. Seal feasibility was defined by the ability to obtain and maintain an airtight seal over the incision and its immediate surroundings without folds or bridging, which depends on anatomical contour, incision length and geometry, and the availability of a customisable rather than a fixed‐format dressing. Intended therapy duration was defined by the planned course relative to the maximum wear time declared for the system. The planned course is a clinical decision, whereas the declared wear time was used only as a technical constraint that the selected system must be able to accommodate; because no indication‐specific evidence on the optimal duration of iNPWT was identified, declared wear time was not treated as a recommendation on how long therapy should be continued. Terms that could not be operationalised in a reproducible way for closed incisions, in particular graded categories of exudate volume and the presence of excavation or cavity, were removed from the framework rather than retained as ambiguous decision nodes.

Framework development was also informed by practical device constraints encountered in routine care at the University Hospital of Padova, a referral centre for complex wound management with approximately 10 years of experience in iNPWT use. To keep the origin of each element transparent, every component of the framework is labelled as direct clinical evidence, indirect clinical evidence, documented technical characteristic, or expert clinical consideration. The corresponding mapping is provided in Table S1. These labels describe the provenance of each statement and are not a graded assessment of certainty.

## 3 Results

### 3.1 From Open Wound NPWT to Closed Incision Platforms

The transition from conventional open wound NPWT to compact incisional systems reflects a change in target rather than a change in biological rationale. Conventional NPWT was developed for open wounds and was associated with fluid evacuation, oedema control, and mechanical effects at the wound interface [1, 2, 3]. In closed incisions, the main target is the incision line and adjacent dead space rather than an exposed wound bed. Modern iNPWT systems combine an adhesive drape with an integrated manifold designed to distribute negative pressure across the incision and surrounding skin. The distinction is operational as well as conceptual. Conventional NPWT requires a filler conformed to a cavity, anticipates larger fluid volumes, and is managed with scheduled dressing changes; incisional systems are applied to intact skin over an apposed suture line, are dimensioned for the incision rather than for a defect, and are intended to remain undisturbed for a declared wear time. A system that is physically capable of being applied to a small open wound is not thereby an incisional device, and the labelled indication of each system should be verified before use [11, 12, 13, 14].

Contemporary systems differ along a small number of axes that are relevant to selection rather than merely to chronology. Pressure generation may be electromechanical, using a battery‐powered pump, or mechanical, using a preloaded spring that maintains pressure without batteries or lead wires [13, 16]. The dressing interface ranges from multilayer absorbent dressings with a silicone contact layer, through soft silicone bordered dressings, to reticulated open‐cell foam bolsters combined with a dedicated skin‐interface layer [11, 12, 13, 14]. Fluid handling is achieved by absorption within the dressing combined with moisture vapour transmission through the outer film, by transfer of exudate into a canister, or by a combination of the two [11, 12, 13, 14]. Canister configuration therefore determines whether fluid accumulates within the dressing or is removed from it, which is relevant because dressing saturation is a documented determinant of the pressure actually delivered at the wound surface. A bench and computational comparison of a canister‐based and a canisterless single‐use system reported that delivered pressure diverged from the nominal setting as the dressing became loaded with fluid, and that this divergence differed between the two design concepts [15]; that work was industry‐funded and compared two systems only, so it establishes fluid loading as a determinant of delivered pressure rather than the clinical superiority of either configuration. Portability, operating noise, and alerting also differ in ways documented in labelling: mechanically powered systems operate silently and require no battery, some electromechanical pumps produce an intermittent cycling sound, and alert modalities range from visual indicators alone to combined visual and audible alarms [11, 12, 13, 14]. Intended wear time is declared per system and per dressing configuration and is influenced by fluid load as well as by the planned clinical course [11, 12, 13, 14].

The characteristics of the systems referred to in this review are summarised in Table 1, whose caption states the provenance and the limits of those entries.

**TABLE 1:** Declared technical characteristics of the negative pressure systems referred to in this review.

Characteristic	PICO (Smith + Nephew)	Prevena (3M/Solventum)	Avance Solo (Mölnlycke)	Snap (3M/Solventum)
Labelled indication	Open wounds and closed surgical incisions	Closed surgical incisions	Closed surgical incisions, open wounds, grafts and flaps	Wound management by application of negative pressure, labelled for small to medium wounds; not presented in labelling as an incision management system
Pressure generation	Battery‐powered electromechanical pump, single use	Battery‐powered electromechanical therapy unit, single use	Battery‐powered electromechanical pump, single use	Mechanically powered preloaded spring; no battery or lead wires
Nominal negative pressure	−80 mmHg, continuous	−125 mmHg, continuous	−125 mmHg, continuous	−75, −100 or −125 mmHg according to cartridge, continuous
Dressing and interface	Multilayer absorbent dressing with silicone adhesive contact layer	Peel‐and‐place dressing for linear incisions of 13 or 20 cm, or customisable dressing; reticulated open‐cell foam bolster with skin interface layer containing 0.019% ionic silver	Multilayer bordered dressing with soft silicone contact layer and borders	Reticulated open‐cell foam wound interface with hydrocolloid sealant layer and integrated port; customisable
Canister configuration	Canister‐free	Canister‐based; 45 mL, or 150 mL with the Prevena Plus therapy unit	Canister‐based; 50 mL	Cartridge integrating the pressure source and fluid containment; 60 mL or 150 mL
Fluid management mechanism	Absorption within the dressing combined with evaporation through the outer film	Fluid wicked from the incision by the interface layer and collected in the canister	Shared between the absorbent dressing and the canister	Exudate drawn into the cartridge and gelled
Declared wear time	Up to 7 days on low exuding wounds with a single‐dressing kit; up to 4 days on moderately exuding wounds unless further dressings are used; up to 14 days for the 14‐day system	Continuous therapy for a minimum of 2 and a maximum of 7 days	Up to 7 days per dressing; system use up to 14 days	Not specified as a fixed limit; determined by dressing change protocol and cartridge capacity
Alerting, noise and power	Visual indicators only; no audible alerts; battery powered	Visual and audible alerts for leak, full canister and low battery; battery powered	Visual and audible alarms for leak, blockage and low battery; intermittent cycling sound, for which a night case is provided	Visual indicator for full cartridge or loss of negative pressure; no audible alarm; silent operation, no battery
Principal documented limitations	Fluid capacity confined to the dressing; declared wear time shortens as exudate increases	Requires canister management; dressing contains metallic silver, which is relevant to diagnostic imaging; fixed maximum therapy duration	Requires canister management; cycling sound may disturb sleep	Labelled for small to medium wounds; cartridge capacity limits use where drainage is greater; not an incision‐specific dressing format

*Note:* Entries are taken from manufacturers' instructions for use, clinician guidelines, and regulatory documentation [11, 12, 13, 14] and are technical specifications, not comparative performance data. No ranking or clinical preference among systems is implied. Where reliable documentation was not identified, the field is reported as not specified.

In this setting, device performance depends on the dressing incision interface. Loss of seal over contoured anatomy, adhesive‐related skin injury, and discomfort with reduced adherence to the intended wear time are relevant practical failure modes. These interface‐related variables influence real‐world effectiveness and should be considered during patient selection and device choice. Compact platforms improve portability and can facilitate outpatient management, but their fluid‐handling capacity and tolerance on fragile skin differ across systems [11, 12, 13, 14].

### 3.2 Evidence Base Across Surgical Settings

The evidence base for iNPWT spans elective surgery, semiurgent procedures, emergency trauma and reconstructive surgery. Meta‐analyses that pooled procedures generally reported a reduction in surgical site infection with iNPWT compared with standard dressings, although certainty and effect size varied according to study design, endpoint definition, follow up duration, and risk of bias [7, 8, 9]. Earlier systematic reviews and GRADE‐based assessments described low certainty evidence and highlighted recurrent limitations such as limited blinding and industry involvement [7, 8]. More recent analyses reported higher certainty for the specific endpoint of surgical site infection, while still acknowledging persistent heterogeneity across procedures and secondary outcomes [9]. A 2026 meta‐analysis of 85 randomised trials confirmed lower risks of overall, superficial, and deep surgical site infection with iNPWT, together with higher risks of skin blistering and device‐related adverse events [29].

Table 2 links the clinical studies cited in this review to the systems they evaluated and can be read against the technical characteristics in Table 1. Most randomised evidence derives from two systems, a canister‐free dressing operating at −80 mmHg and a canister‐based foam dressing operating at −125 mmHg, and trials of each system include both positive and neutral results [17, 18, 19, 20]. The only randomised comparison between two commercial systems identified, a single‐centre three‐arm trial in emergency laparotomy, found that both reduced surgical site infection compared with conventional dressings without differing from each other, but it analysed 123 patients and was not powered for the inter‐device comparison [21]. A retrospective claims‐database comparison publicised by the manufacturer of one system favoured that system [22], whereas an independent meta‐analysis of randomised trials in abdominal surgery reported comparable reductions in surgical site infection with both systems [30]. The clinical evidence can therefore be linked to named systems, but it does not show that one system outperforms another.

**TABLE 2:** System‐specific summary of the clinical studies of primarily closed incisions cited in this review.

Study	Design; n	Setting	System; nominal pressure; fluid management	Comparator	Main endpoint	Principal result	Device‐related adverse events
Hyldig 2019 [17]	Multicentre RCT; 876	Caesarean section, pre‐pregnancy BMI ≥ 30 kg/m^2^	PICO; −80 mmHg; canister‐free	Standard dressing	SSI requiring antibiotics at 30 days	SSI 4.6% versus 9.2% (RR 0.50, 95% CI 0.30 to 0.84); less exudate; no difference in dehiscence	NR
Gillespie 2021 [18]	Multicentre RCT; 2035	Caesarean section, BMI ≥ 30 kg/m^2^, elective or semi‐urgent	PICO; −80 mmHg; canister‐free	Standard dressing	Cumulative SSI	SSI 7.4% versus 9.7% (RR 0.76, 95% CI 0.57 to 1.01)	Blistering 4.0% versus 2.3% (RR 1.72, 95% CI 1.04 to 2.85)
Kwon 2018 [19]	Single‐centre RCT; 119 incisions	Elective vascular surgery, high‐risk femoral incisions	Prevena; −125 mmHg; canister‐based	Standard gauze	Major wound complications at 30 days	8.5% versus 25%; reoperation 8.5% versus 18.3%; readmission 6.8% versus 16.7%	NR
Lenselink 2026 [20]	Multicentre RCT; 290	Arterial surgery with longitudinal groin incision	Prevena; −125 mmHg; canister‐based	Standard surgical dressing	Wound complications (infection, seroma, necrosis, dehiscence) at 30 days	No reduction in overall wound complications	NR
Costa MJ 2024 [21]	Single‐centre three‐arm RCT; 123 analysed	Emergency laparotomy	Prevena (−125 mmHg; canister‐based) and PICO (−80 mmHg; canister‐free)	Conventional dressing	SSI at 30 days	SSI 12.2% (Prevena), 7.7% (PICO), 30.2% (control); no difference between systems	NR
Bashyal 2025 [22]	Retrospective claims‐database cohort; 57 181	Orthopaedic and cardiovascular surgery	PICO (−80 mmHg) versus Prevena (−125 mmHg)	Other system	Surgical site complications, length of stay, cost	Lower odds of dehiscence and lower resource use with PICO	NR
Abesamis 2019 [23]	Prospective consecutive cohort; 16	Abdominoplasty	Prevena Plus; −125 mmHg; canister‐based	Standard dressing	Drain output; time to drain removal	370 versus 1269 mL; drain removal 5.3 versus 10.6 days	None observed in either group
Facchin 2021 [24]	Retrospective case–control; 26	Postbariatric brachioplasty	PICO; −80 mmHg; canister‐free; 7 days	Dry dressing	Dressing changes, time to dry, length of stay, complications	2.0 versus 4.9 dressing changes; time to dry 9.4 versus 17.7 days; stay 3.1 versus 5.3 days	Blistering in 1 patient per group; hyperchromic scar at 90 days in 7/14 versus 0/12
Stannard 2012 [25]	Multicentre RCT; 249 patients, 263 fractures	High‐energy tibial plateau, pilon and calcaneal fractures	V.A.C. (KCI), conventional NPWT unit over the incision; canister‐based; pressure NR	Standard dressing	Infection; dehiscence	Infection 10% versus 19%; dehiscence 8.6% versus 16.5%	NR
Costa ML 2020, WHIST [26]	Multicentre RCT; 1548	Lower‐limb fractures associated with major trauma	iNPWT; system NR	Standard dressing	Deep SSI at 30 days	5.8% versus 6.7%, NS; at 90 days 11.4% versus 13.2%	NR
Timmermans 2022 [27]	Within‐patient RCT	Mastectomy	PICO; −80 mmHg; canister‐free	Standard dressing	Scar quality; patient‐reported outcomes	Patient‐reported scar outcomes differed; objective scar measures not consistently improved	NR

*Note:* The system is given as named in each publication; nominal pressure and fluid‐management configuration are the declared properties of that system (Table 1) and are not adjustable in commercial incisional systems. Fields not stated in the primary publication are reported as NR. Results are given as intervention versus comparator unless otherwise stated.

Abbreviations: BMI, body mass index; CI, confidence interval; NR, not reported; NS, not significant; RCT, randomised controlled trial; RR, risk ratio; SSI, surgical site infection.

### 3.3 High Risk Closed Incisions and Prophylaxis

Patient selection in prophylactic iNPWT studies is largely driven by factors known to modify incision healing and infection risk, including obesity, diabetes, smoking, immunosuppression, peripheral arterial disease, malnutrition, steroid therapy, and prior radiotherapy [10]. In obese women undergoing caesarean section, a pragmatic randomised clinical trial reported a reduction in surgical site infection with prophylactic iNPWT compared with standard dressings [17]. A large multicentre randomised trial in the same setting reported a directional reduction in infection that did not reach significance in the primary analysis and identified a higher incidence of skin blistering in the iNPWT group [18]. These data suggest that prophylaxis may shift the spectrum of complications, reducing some events while introducing others. Blistering at the dressing margin is a recognised form of medical adhesive‐related skin injury, arising when an adhesive is applied under tension or when shear develops between a comparatively rigid dressing and mobile skin, and it is classified together with epidermal stripping and skin tears among the mechanical adhesive injuries [28]. It is particularly relevant to iNPWT because the perilesional skin is deliberately included within the sealed field and is subject to sustained negative pressure as well as to tension at the dressing border. Epidermolysis at this site converts a primarily closed incision into an open wound requiring additional care and may terminate the therapy that was intended to protect it. Susceptibility is increased in atrophic or oedematous skin, in previously irradiated fields, and in patients on long‐term corticosteroid or immunosuppressive therapy [28]. Adhesive selection, application without stretch, avoidance of folds, atraumatic removal technique, and early reassessment of the dressing margins are therefore practical components of safe iNPWT use, and perilesional skin tolerance is treated as an explicit node in the decision framework.

In vascular surgery, a randomised clinical trial in high risk groin incisions reported fewer major wound complications, reoperations, and readmissions with negative pressure therapy than with standard dressings [19]. A meta‐analysis focused on vascular groin wounds also reported lower surgical site infection rates with closed incision NPWT, with greater benefit in studies with higher baseline infection rates in the control group [31]. A subsequent multicentre randomised trial of a canister‐based system in arterial groin incisions found no reduction in overall wound complications [20].

In plastic and reconstructive surgery, studies in abdominoplasty and postbariatric body contouring reported reductions in local wound complications in selected patients, with several analyses focusing on seroma burden and drain dependence rather than infection alone [23, 24]. These data were generated in clinically heterogeneous populations and with nonuniform endpoints.

### 3.4 Emergency Trauma and Flap Related Incisions

In flap surgery, early postoperative oedema, shear, and venous congestion may threaten perfusion and predispose to marginal necrosis. Many emergency patients also have fragile skin or receive anticoagulant therapy. Where iNPWT is applied over an incision in this setting, the relevant requirements are perilesional skin tolerance, the ability to inspect the incision and its margins, and predefined criteria for discontinuation; no direct comparative evidence supports a specific pressure setting or canister configuration in this population. These considerations support conservative selection in emergency reconstruction, careful attention to haemostasis at closure, and predefined discontinuation criteria when pain, bleeding, or skin injury occurs.

Evidence in trauma related incisions was mixed. In a prospective randomised multicentre trial of high risk lower extremity fractures, incisional NPWT was associated with fewer infections and less dehiscence than standard dressings [25]. In contrast, the WHIST randomised clinical trial in major trauma related lower limb fractures found no significant reduction in deep surgical site infection at 30 or 90 days with iNPWT compared with standard wound dressing [26].

### 3.5 Boundary With Conventional NPWT for Open Wounds

The framework does not extend to open wounds. Chronic ulcers, cavities, and lesions with substance loss differ from primarily closed incisions in wound biology, in the requirement for a filler interface, and in the volume and duration of drainage, and they are addressed by conventional NPWT rather than by incisional platforms. The boundary matters in practice because compact single‐use systems are physically capable of being applied to small open wounds, and some carry labelling for that use, which can obscure the distinction between the two indications.

The evidence supporting NPWT in chronic lower extremity wounds is limited and condition specific. A Cochrane review on NPWT for leg ulcers concluded that rigorous randomised evidence was scarce and that applicability was restricted by the contexts studied [32]. In a multicentre randomised comparative efficacy trial, a single use NPWT system was clinically comparable to traditional NPWT in selected venous leg ulcers and diabetic foot ulcers within a noninferiority design based on changes in ulcer dimensions over 12 weeks [33]. These data concern open wound management and are not transferable to closed incisions; they are cited here to delimit the scope of the present framework rather than to support an incisional indication. Figures 1 and 2 illustrate applications of portable negative pressure systems to open wounds and are shown for the same purpose.

**FIGURE 1:**
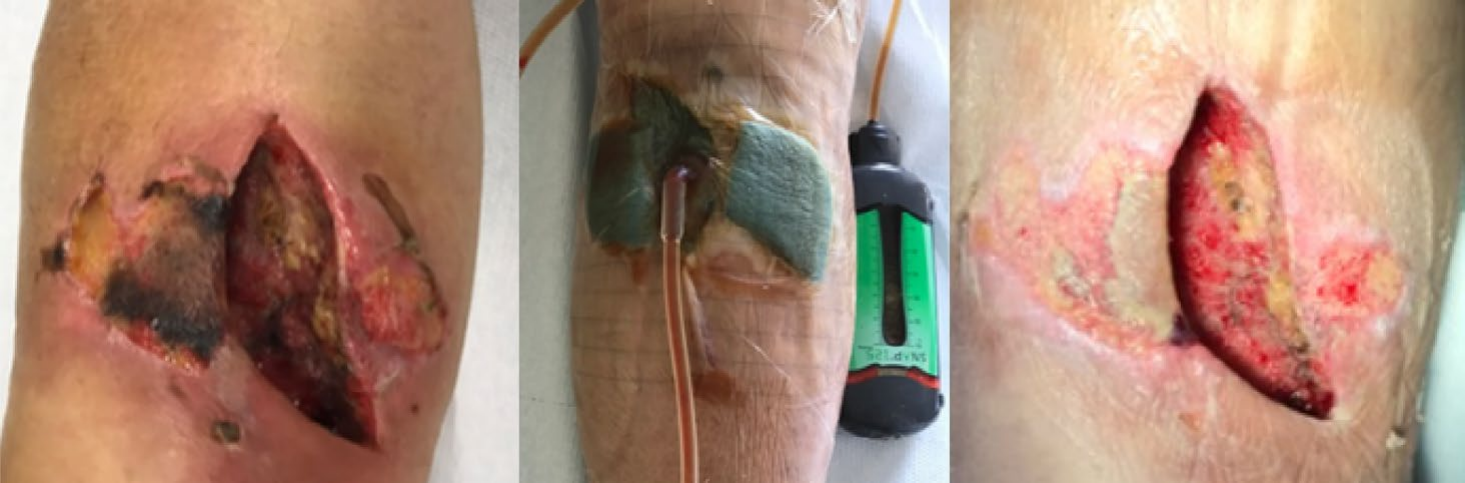
Application of a portable single‐use negative pressure system (Snap Therapy System, 3M/Solventum) to a post‐traumatic wound with substance loss in the posterior region of the leg following a motorcycle accident. This is conventional open wound NPWT and lies outside the scope of the incisional framework; the image is shown to illustrate the boundary between the two indications.

**FIGURE 2:**
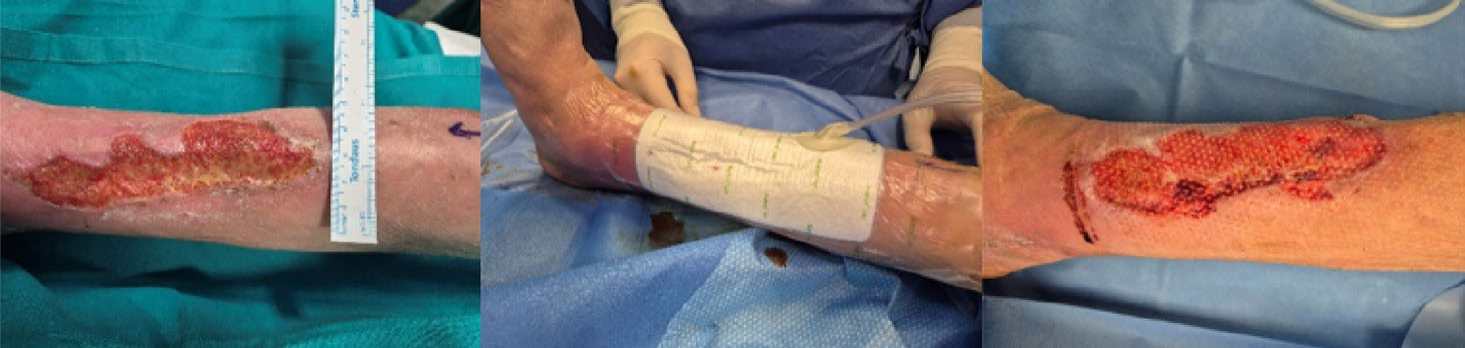
Patient with chronic leg ulcer affected by rheumatoid arthritis on cortisone therapy at the first dressing change after 1 week of negative pressure therapy (Avance Solo, Mölnlycke). As in Figure 1, this is an open wound application shown to delimit the scope of the incisional framework; it also illustrates the perilesional skin fragility that determines the adhesive interface requirement.

### 3.6 Device Attributes, Scar Outcomes, and Health Economics

The resulting decision framework is shown in Figure 3. It begins with confirmation that the wound is a primarily closed incision and that patient and procedural risk justify iNPWT, with bleeding risk and other contraindications acting as gating conditions before any device is considered. It then proceeds through four requirement nodes: anticipated fluid‐handling requirement, perilesional skin tolerance, seal feasibility over the relevant anatomy, and intended therapy duration with the associated mobility requirement. Each node resolves into a device requirement rather than into a named product: active fluid collection or dressing‐based fluid management; a low‐trauma silicone interface or a standard adhesive; a customisable or a fixed‐format dressing; a system whose declared wear time covers the planned course. Commercially available systems appear only as examples of configurations that satisfy a given requirement, and their position in the figure does not indicate preference. Nominal pressure is not used as a decision node, because the reviewed clinical literature does not isolate pressure from the dressing and pump architecture in which it is delivered, and because the pressure effectively delivered at the wound surface also depends on the fluid load of the dressing [15]. The provenance of each element of the framework is given in Table S1.

**FIGURE 3:**
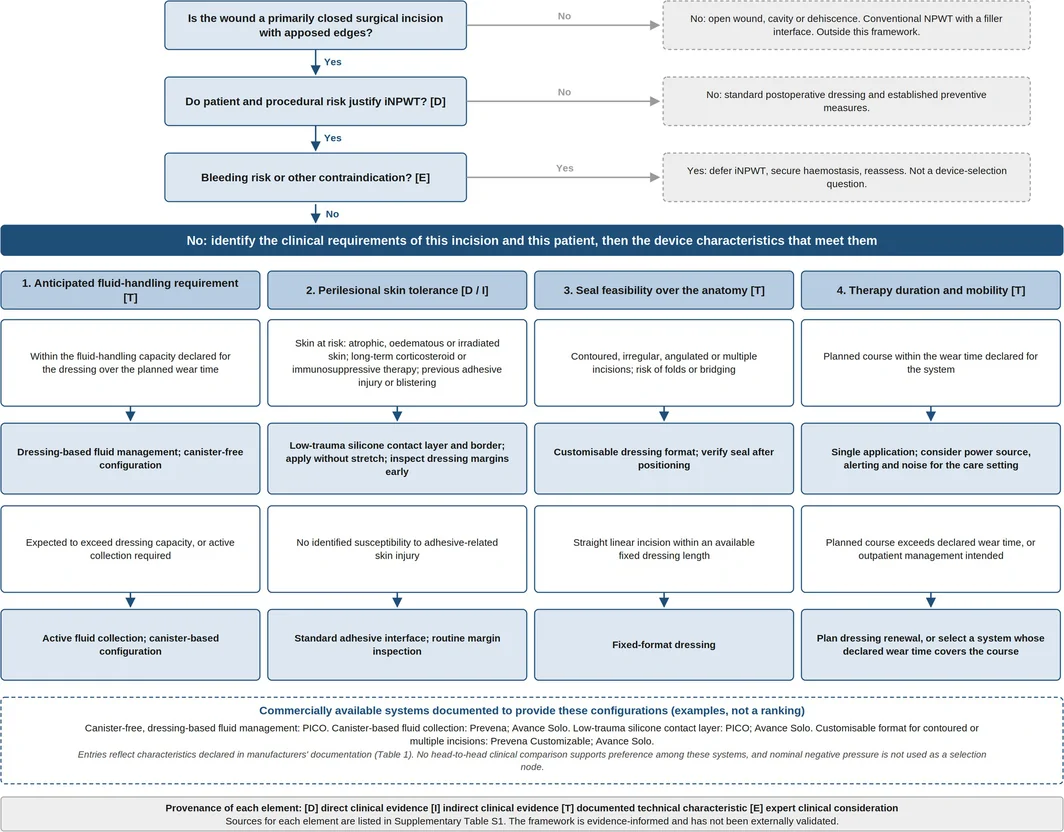
Clinical‐requirement‐first decision framework for incisional negative pressure wound therapy device selection. The framework is entered only for primarily closed surgical incisions in patients whose risk profile justifies iNPWT; bleeding risk and other contraindications act as gating conditions. Four requirement nodes are then resolved into device requirements: anticipated fluid‐handling requirement, perilesional skin tolerance, seal feasibility over the relevant anatomical contour, and intended therapy duration with the associated mobility requirement. Commercially available systems are shown only as examples of configurations satisfying a given requirement and are not ranked. Provenance labels indicate whether an element derives from direct clinical evidence (D), indirect clinical evidence (I), a documented technical characteristic (T), or expert clinical consideration (E); sources are listed in Table S1. The framework is evidence‐informed and has not been externally validated. iNPWT, incisional negative pressure wound therapy; NPWT, negative pressure wound therapy.

Scar‐related outcomes were reported in a separate systematic review, which found moderate level evidence suggesting reduced scar width and improved patient reported and observer reported scar satisfaction with iNPWT [34]. In a within patient randomised trial in mastectomy incisions, differences were observed in patient reported scar outcomes between iNPWT and standard care, whereas objective scar measures were not consistently improved [27] (Figure 4).

**FIGURE 4:**
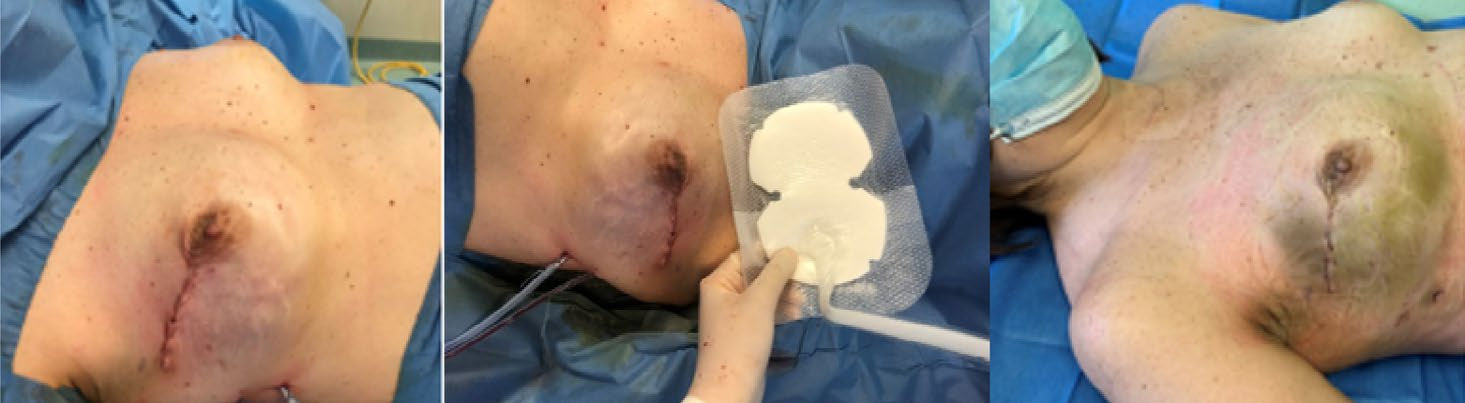
Application of iNPWT (PICO device Smith and Nephew) in a patient with breast cancer who underwent radiotherapy, mastectomy and immediate reconstruction with implant. Previously irradiated skin is a determinant of perilesional skin tolerance within the framework.

Economic evaluations also differed across settings. A trial based economic evaluation alongside a pragmatic caesarean section trial reported that iNPWT was likely cost saving and more effective from a short term healthcare perspective, with the largest contribution in higher body mass index strata [35]. In another health system, a separate economic evaluation concluded that routine use of closed incision NPWT in obese women undergoing caesarean section was unlikely to be cost‐effective, with substantial uncertainty around incremental costs and effects [36]. Cost therefore cannot be ranked across systems in a way that transfers between health systems, because acquisition price, procurement arrangements, reimbursement, and the cost of unplanned dressing changes differ by country and by institution. Practical characteristics documented in labelling, such as the absence of batteries and audible alarms in mechanically powered systems or the intermittent cycling sound of some electromechanical pumps, are relevant to tolerability and to selection in specific care settings, but no comparative clinical data link them to wound outcomes [11, 12, 13, 14].

An updated meta‐analysis of randomised controlled trials in laparotomy reported a protective effect of prophylactic NPWT against surgical site infection and also highlighted the influence of small study effects [37]. Across the reviewed literature, comparative evidence rarely isolated pressure as an independent determinant of outcome because pressure settings were embedded within proprietary dressing‐manifold‐pump systems. Commercial incisional systems typically deliver fixed pressures that are a property of the system rather than an adjustable setting, and planned wear time is also influenced by dressing capacity, canister use, and battery endurance rather than by incision biology alone. Most comparative studies should therefore be interpreted as evaluations of complete systems rather than as definitive tests of specific pressure values, and identical nominal pressure across systems should not be read as evidence of equivalent performance.

## 4 Discussion

This review supports a selective and risk based use of iNPWT. The available literature suggests that the intervention is most relevant when baseline risk of wound complications is sufficiently high and when incision level mechanisms are plausible targets of treatment, particularly dead space, oedema, tension, or superficial fluid accumulation. This principle is consistent across caesarean section, vascular groin surgery, laparotomy, and selected reconstructive procedures, although the magnitude and certainty of benefit differ by indication [6, 7, 8, 9, 17, 18, 19, 23, 24, 31, 37].

The reviewed studies also show that iNPWT should be considered an adjunct rather than a substitute for standard preventive measures. Procedure‐specific benefit depends on the interaction between patient risk profile, local tissue condition, and the technical limitations of the selected device. Device‐related adverse events remain clinically relevant. Skin blistering is a consistent concern in prophylactic use over fragile or irradiated skin, and excessive suction over inadequately controlled raw surfaces may aggravate bleeding. Leakage, maceration, and poor seal stability can negate potential benefit, particularly when drainage exceeds the fluid‐handling capacity declared for a canister‐free configuration. Skin preparation, careful placement of adhesive borders, and minimisation of folds are practical measures that may reduce leakage and blistering. In high‐risk elective surgery, coordination with drains and incision geometry is also important to preserve seal integrity and maintain consistent negative pressure over the wound.

The emergency setting requires particular caution. The discrepant findings between the lower extremity fracture trial by Stannard et al. and the WHIST trial suggest that deep infection after major trauma is not determined by incision management alone [25, 26]. Fracture severity, contamination, fixation strategy, and systemic injury burden may outweigh any superficial dressing effect. For this reason, the framework treats emergency indications as selective and applies the same requirement‐based logic, since the features that differ in this population are skin tolerance, observability of the incision, and bleeding risk rather than the indication itself.

Open wounds fall outside the scope of incisional therapy. The current evidence does not support extension of incisional platforms to chronic wound care, and a lesion that behaves as an open wound with a cavity and sustained drainage should be managed with conventional NPWT, which retains the stronger direct evidence base in that setting [32, 33]. Maintaining this boundary is also what allows a device‐selection framework for closed incisions to remain internally coherent, since cavity management and filler selection are requirements of a different therapeutic problem (Figure 5).

**FIGURE 5:**
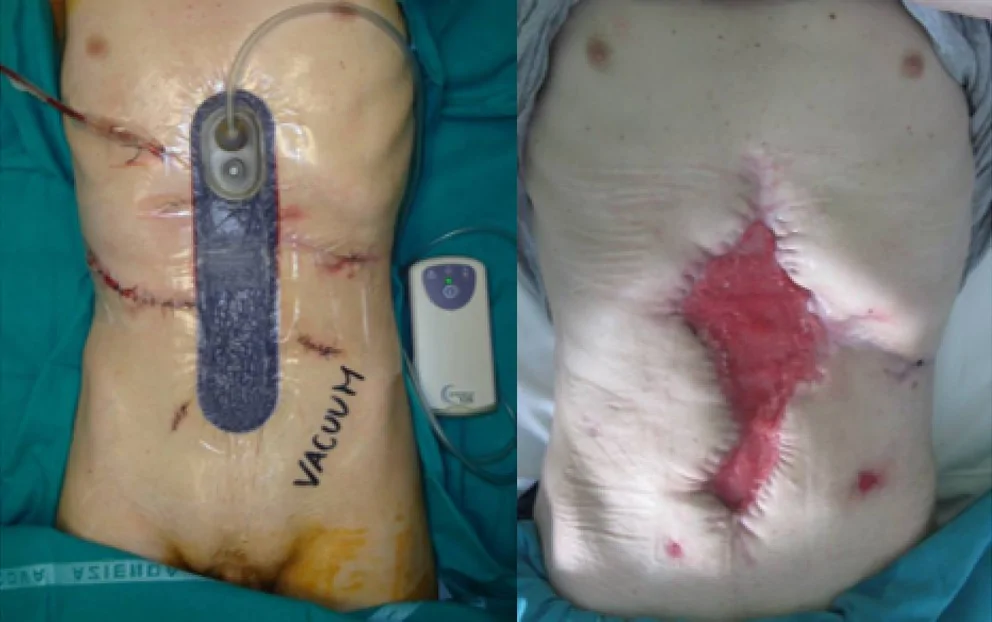
Dehiscence following midline laparotomy (3M Prevena device). Once dehiscence has occurred, the wound is no longer a primarily closed incision, and management follows conventional open wound NPWT principles; the image is shown to illustrate this transition.

The contribution of the present work is not the observation that iNPWT should be used selectively, which has been established since the 2017 international consensus and is supported by subsequent meta‐analytic evidence [6, 7, 8, 9]. It is the organisation of documented device characteristics around clinical requirements that can be assessed at the bedside. The question of which incisions and which patients may benefit is addressed by existing indication‐based recommendations; the question of which system characteristics are relevant once iNPWT has been judged appropriate is not, and that is the question the present framework addresses. The framework does not function as a validated predictive model and should not be interpreted as proof of effectiveness. It was derived from a narrative synthesis of heterogeneous studies, from declared technical characteristics, and from practical device constraints encountered in routine care. The present work did not include aggregated outcome data from a local cohort and therefore cannot support claims that use of the framework reduces complications or accelerates healing.

Several limitations should be acknowledged. The literature search was targeted rather than systematic and was restricted to a single database, so relevant studies indexed elsewhere may have been missed; screening was not duplicated and no formal quality appraisal was undertaken. Endpoint heterogeneity remains substantial. Studies variably reported superficial or deep surgical site infection, seroma, dehiscence, drain duration, blistering, pain, and patient reported outcomes. Study quality was inconsistent, blinding was often limited, and manufacturer involvement was frequent in this field [7, 8, 9, 27, 34, 35, 36, 37]. The only randomised comparison between commercially available incisional systems was small and not powered for that comparison [21], and published studies evaluate complete proprietary systems rather than isolated technical variables such as pressure level, dressing interface, or canister configuration. Identical nominal negative pressure should therefore not be taken to imply biomechanical or clinical equivalence between proprietary systems, because the pressure and the mechanical effect transmitted at the incision depend on the complete pump, tubing, and dressing architecture and on the fluid load of the dressing [15, 16]. A comparable limitation applies to treatment duration. No study identified in this review compared different durations of iNPWT within the same indication; duration was fixed by the study protocol or by the wear time of the system, ranging from a mean of 2.5 days in the fracture trial by Stannard et al. to 7 days in other settings [24, 25], and the application period of 7 days referred to in the 2017 international consensus reflects expert agreement rather than comparative data [6]. Manufacturer‐declared wear time defines how long a given system may technically remain in place and enters the framework as a technical constraint, not as an evidence‐based recommendation on treatment duration. For these reasons several components of the framework are evidence‐informed rather than externally validated, and it should be used as structured decision support rather than as a prescriptive algorithm. Future trials should separate superficial from deep endpoints and report unplanned dressing changes, concomitant drain use, perilesional skin protection, and reasons for premature discontinuation.

Future studies should prospectively validate the proposed framework in the two domains addressed here: prophylaxis in high‐risk elective incisions, and emergency trauma and flap‐related incisions. Standardised reporting of dressing configuration, actual treatment duration, concomitant drain use, reasons for premature discontinuation, and adverse events related to the device would improve reproducibility and allow more meaningful comparison across studies. Trials comparing different durations of therapy within the same indication would be required before an evidence‐based treatment duration could be defined. In parallel, standardised bench comparison of complete systems under matched conditions, using predefined endpoints such as pressure delivered at the wound surface, pressure stability under progressive fluid loading, and wound‐edge traction, would supply the head‐to‐head technical evidence that the clinical literature currently cannot provide [15, 16]. Such testing constitutes a separate experimental investigation with its own protocol and validated models, and its results would be required before any hierarchy among commercial systems could be proposed. Health economic evaluation should be embedded prospectively and interpreted as procedure specific and health system specific rather than assumed to be transferable across settings. Comparative evaluation of canister‐free and canister‐based systems, along with procedure‐specific health economic analyses, remains necessary before wider implementation can be recommended.

## 5 Conclusion

Incisional negative pressure wound therapy should be used selectively as an adjunct to standard wound prevention strategies rather than as a routine intervention. Its clinical value appears most plausible in high‐risk closed incisions in which dead space, oedema, tension, or superficial fluid accumulation are relevant contributors to wound morbidity. Once its use has been judged appropriate, device selection should be organised around the clinical requirements of the incision and the patient, namely anticipated fluid‐handling requirement, perilesional skin tolerance, seal feasibility, and intended therapy duration, matched to the documented characteristics of the available systems. Current evidence does not establish a hierarchy among commercially available incisional systems, and the proposed framework should be regarded as structured decision support requiring prospective validation rather than as a validated algorithm.

## Funding

The authors have nothing to report.

## Ethics Statement

The authors have nothing to report.

## Consent

The authors have nothing to report.

## Conflicts of Interest

The authors declare no conflicts of interest.

## Supporting information


**Table S1:** Provenance of the elements of the decision framework shown in Figure 3. Labels describe the origin of each statement and are not a graded assessment of certainty.

## Data Availability

The data that support the findings of this study are available from the corresponding author upon reasonable request.
