# Supplementary material for: Current Incisional Negative Pressure Wound Therapy Systems: Technical Characteristics and a Decision Framework for Device Selection
Source: Int Wound J. 2026 Oct 2;23(10):e71074. doi: 10.1111/iwj.71074 (PMC13633450; doi:10.1111/iwj.71074)
Supplement: Supplementary file 1 [file IWJ-23-e71074-s001.docx]

Supplementary Table S1. Provenance of the elements of the decision framework shown in Figure 3. Labels describe the origin of each statement and are not a graded assessment of certainty.

| **Framework element** | **Provenance** | **Basis** |
| --- | --- | --- |
| Entry criterion: primarily closed surgical incision with apposed edges | Direct clinical evidence | Randomised trials and meta-analyses of iNPWT were conducted over primarily closed incisions (7,8,9,11,12,13,14,17,18,25) |
| Patient and procedural risk profile justifies iNPWT | Direct clinical evidence | Consensus recommendations and trials selecting high-risk populations (6,10,11,12,13,14) |
| Greater absolute benefit where baseline complication risk is higher | Indirect clinical evidence | Subgroup and meta-analytic findings in vascular groin wounds and caesarean section (12,14,23) |
| Anticipated fluid-handling requirement determines canister-based versus dressing-based management | Documented technical characteristic | Declared fluid-handling capacity, canister volumes and wear times (29-32); not provided in comparable terms for all systems, so the criterion is qualitative |
| Dressing saturation may reduce the pressure delivered at the wound surface | Indirect evidence from bench and computational testing | Comparison of a canister-based and a canisterless single-use system (26); industry-funded, two systems only |
| Perilesional skin tolerance determines the adhesive interface requirement | Direct clinical evidence for the adverse event; indirect for the interface choice | Higher incidence of blistering with iNPWT in a multicentre randomised trial (12); classification and risk factors for medical adhesive-related skin injury (28) |
| Seal feasibility determines the need for a customisable rather than a fixed-format dressing | Documented technical characteristic | Declared dressing formats, incision length limits and customisable options (29-32) |
| Intended therapy duration must be covered by the declared wear time | Documented technical characteristic | Declared maximum wear times per system and per dressing configuration (29-32); technical constraint only, as no comparative evidence on optimal treatment duration was identified (6,16,17) |
| Bleeding risk and other contraindications as gating conditions | Expert clinical consideration | Contraindications and warnings in device labelling (29-32); no comparative clinical data identified |
| Portability, operating noise, alerting and power source as tolerability considerations | Documented technical characteristic | Declared power source, alarm modality and operating characteristics (27,29-32) |
| Predefined criteria for discontinuation on pain, bleeding or skin injury | Expert clinical consideration | Practice at the authors' institution; not derived from comparative data |
| Absence of a hierarchy among commercially available incisional systems | Stated limitation | One small randomised comparison of two systems without an inter-device difference (35); no adequately powered head-to-head trial |
